# Demographic Assessment of Down Syndrome: A Systematic Review

**DOI:** 10.3390/ijerph18010352

**Published:** 2021-01-05

**Authors:** Agustín Huete-García, Mónica Otaola-Barranquero

**Affiliations:** Institute on Community Integration (INICO), School of Education, University of Salamanca, Paseo de Canalejas 169, 37008 Salamanca, Spain

**Keywords:** Down Syndrome, demography, assessment, incidence, prevalence, rights, policies and programs, intellectual disability

## Abstract

The objective of this study is to assess the evidence about the demographic transformation of the Down Syndrome population, with a specific focus on prenatal testing, and to identify sources frequently used for demographic assessment of Down Syndrome in the world. We reviewed existing studies on demographic transformations in the population with Down Syndrome, specifically birthrate indicators, under the Preferred Reporting Items for Systematic Reviews and Meta-Analyses (PRISMA) Statement. The searches were made in Medline (via EBSCO Host), Academic Search Complete (via EBSCO Host), PsycINFO (via EBSCO Host), Web of Science (Core Collection), Public Health Database (via ProQuest), and The Cochrane Library. The terms were developed through Medical Subject Headings (MESH) and American Psycological Asociation Thesaurus of Psychological Index Terms (APA). Full texts were reviewed if information was given regarding location and birthrate for a range of three years or more, and if the first and last year considered was within 1960 and 2019. We found 22 references with a period of study between 1960 and 2019 following the global spread of prenatal testing for Down Syndrome. We found a consistent association between prenatal diagnosis and birthrate, enough to explain the significant fall in the prevalence of Down Syndrome, a somewhat rising incidence of Down Syndrome related to increased maternal age and extension of fertility services in healthcare systems, a generalized use of specific congenital birth defect registries as the primary source of data, and an unclear influence of socio-cultural and territorial variables. Our findings can inform research, policy, and practice to improve the reproductive health and quality of life of the population with Down Syndrome.

## 1. Introduction

Down syndrome (DS) is the most common genetic abnormality associated with a varying degree of intellectual disability, some health and developmental effects, and peculiar physical features that give those with it a recognizable appearance. The causes that can explain this genetic alteration are mostly unknown, although it is well recognized that a high incidence occurs with increasing maternal age.

Demographic assessment is widely used in policy-making processes related to social protection, health care, and other public policies worldwide, mainly when there are significant differences in practices across different countries [1]. In this work, we make a demographic evaluation of the population with DS based on a key demographic indicator of incidence: birthrate as the number of infants with DS per 10,000 live births.

There are some hints on how the population with DS may be undergoing a major demographic transformation based on declining birthrates. This situation presents differences by countries that seem related, intuitively, to the influence of cultural factors, physician practices, and women’s decision-making regarding prenatal screening and diagnosis for DS [2].

The origins of prenatal diagnostic techniques began in 1955 [3]. From that moment, multiple methods for the diagnosis of chromosomal abnormalities followed each other [4]. In 1970, amniocentesis was able to diagnose DS with a high degree of certainty, and from then, the technique was spread rapidly in healthcare systems [5].

In 1984, the World Health Organization recommended the use of prenatal diagnostic techniques in all families at risk [6]. The incorporation of prenatal testing has resulted in promoting these techniques as part of the obstetric surveillance and screening programs [7]. Since 2011, non-invasive prenatal testing techniques have been developed and offered as an alternative to invasive testing to eliminate the risk of fetal loss [8].

After five decades of prenatal diagnosis, there is an intense debate about whether the widening and improvements in genetic counseling have resulted in a relative impact on the birth prevalence of DS [9,10,11] and may trigger a significant transformation in the demography of this population.

In the framework of the health-related domains of the International Classification of Functioning, Disability and Health (ICF) [12] and Convention on the Rights of Persons with Disabilities (CRPD) [13], the Disability Rights Movement has become increasingly vocal that prenatal diagnosis applied to DS could be considered discrimination against the disability [14,15].

The objectives of this study are (1) to weigh the evidence about the demographic transformation of the DS population trough birthrate, (2) to analyze factors likely to explain this demographic transformation with a specific focus on prenatal testing, and (3) to identify sources frequently used for demographic assessment of DS in the world. A systematized review was made under the Preferred Reporting Items for Systematic Reviews and Meta-Analyses (PRISMA) Statement to collect data on demographic transformations in the population with DS, specifically birthrate indicators.

Ultimately, the overall goal of the article is to offer critical information to determine the extent to which the demographic transformations of the population with DS around the world are debatable in light of compliance with the International Convention on the Rights of Persons with Disabilities (CRPD), and what implications they have for related public policies.

## 2. Materials and Methods

The review followed the Preferred Reporting Items for Systematic Reviews and Meta-Analyses (PRISMA) Statement. The aim of the screening was to capture all the relevant studies concerning the demographic transformations in the population with DS, specifically over birthrate indicators.

### 2.1. Search Strategy

We searched six databases: Medline (via EBSCO Host), Academic Search Complete (via EBSCO Host), PsycINFO (via EBSCO Host), Web of Science (Core Collection), and Public Health Database (via ProQuest). Search strategies and key words were developed based on Medical Subject Heading (MeSH) and APA Thesaurus of Psychological Index Terms and published search strategies (Table A1 in Appendix A). Additional relevant studies were hand-searched, and previous review articles were used for identification of studies not retrieved through the electronic database.

The authors developed the search strategy (Agustín Huete and Mónica Otaola), and one applied the search (Mónica Otaola). Terms outside from thesaurus are used if it was used during the last decades. The search strategy was based on the MESH and APA Thesaurus. The search used the following key terms based on the main aim of the study: (1) Down Syndrome Population: “Down Syndrome” (MESH), “Down’s Syndrome” (APA), “Trisomy 21”; (2) Demography: “Demography” (MESH), “Epidemiology” (MESH) (APA), “Incidence,” “Birthrate”; and (3) Prenatal testing: “prenatal diagnosis” (MESH) (APA), “prenatal screening,” “prenatal test.”

The review was developed in literature published between 1980 and 2019, as the birthrate prior to the 1970s was assumed to be unaffected by prenatal diagnosis [16]. There were no restrictions on the regions under study. The search only considered articles published in peer-reviewed journals in English and Spanish languages. Boolean operators (and, or, proximity) were used to construct and refine the search.

### 2.2. Inclusion Criteria

Sources retrieved from the six databases were selected for a full-text review if their title or abstract included explicit terms related to Down Syndrome and Demography. The criteria to avoid the risk of bias and choosing the literature were accomplished in the subsequent three phases: (1) title review, (2) abstract review, and (3) full-text review. The records were screened by the authors. Full texts were reviewed if information was given regarding location (city, region, country) and birthrate for a range of three years or more, and if the first and last year considered was within 1960 and 2019.

We excluded: (1) duplicate publications; (2) articles with estimated data; (3) periods under study of less than three years; (4) studies with no quantitative data or that were not convertible to cases per 10,000 births; (5) studies with few cases or small samples; (6) clear region of study. In the case of two or more studies focusing on the same region, we selected the one that provided the most accurate data or the broadest range of years. We contacted the authors whose articles were not available in the database to request the full text. Only one responded and sent us the full article. The total missed articles was four.

### 2.3. Screening and Selection Process

**Figure 1:**
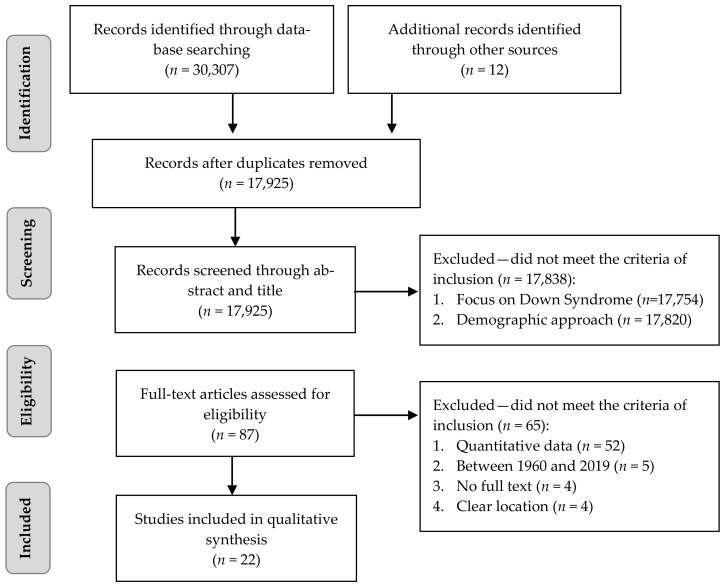
Preferred Reporting Items for Systematic Reviews and Meta-Analyses (PRISMA) Statement flowchart of the study identification, screening, and selection process.

A total of 30,319 sources were found through electronic database searching (*n* = 30,307) and additional relevant studies were found by hand-searching (*n* = 12). After duplicates were removed, a total of 17,925 articles were found. After reviewing titles and abstracts, 87 were assessed for eligibility, in accordance with the aim topics: Down Syndrome (or Trisomy 21) and Demography (or Epidemiology, or Incidence, or Birthrate). In the end, 22 full-text articles met all the inclusion criteria (clear location, quantitative data, and three or more-year period between 1960 and 2019). Figure 1 provides an outline of the search for the studies.

### 2.4. Quality Measurement

We developed a scale to ensure quality and consistency for our review in the last step of the selection process (eligibility). The articles were ranked with scoring criteria, and the articles were blindly reviewed. The scoring criteria were: 2 points if it clearly meets all inclusion criteria, 1 point if it meets all inclusion criteria but not clearly, and 0 points if it was not eligible. To avoid risk of bias in selection, only articles that were ranked with 2 points by both authors were included, and when there was disagreement, it was resolved by consensus. The authors agreed to 81% of the articles. Agreement on those to be included reached 86% and those to be excluded reached 71%.

### 2.5. Data Analysis

Our data analysis is a systematic narrative and descriptive analysis, presented in the text and tables to summarize and explain the characteristics and findings of the included studies. The descriptive analysis includes measures of central tendency (mode, median, and mean) applied to the period’s birthrates included in each study. The change in birthrate was calculated in percentages from the start point to the endpoint, classified as ascent (change positive), descent (change negative), or unclear (change near 0). The birthrate data were converted to a total per 10,000 births. We classified region, source of data (registry, record, or sample). The sources were classified as: (1) Registry (specific population-based congenital defect database), (2) Record (use of files of hospitals, laboratories, other medical centers, other institutions, or death certificates), (3) Survey (developed its own sample for the study); and the principal influence on birthrate when it was clearly recognized in the text. All data were entered and analyzed in a Microsoft Excel database (Microsoft Office Professional Plus 2016) (Microsoft Corporation, Redmond, WA, USA).

## 3. Results

As is show in Table 1, we included 22 studies in this systematic review. The selected studies were classified in accordance with the objectives of this review: (1) main results of the trend in birthrate, (2) main cause of this trend, and (3) main source of the study.

**Figure 2:**
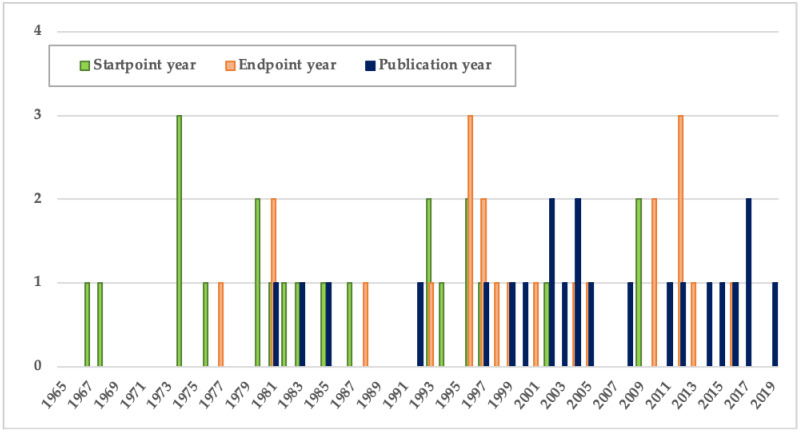
Number of selected studies by year of start point, endpoint, and publication.

The references considered in this systematic review were published between 1980 and 2019, in accordance with the global spread of prenatal testing for DS. We found two studies (9%) with a start point in the 1960s, four studies (18%) with a start point in the 1970s, seven studies (32%) with a start point in the 1980s, and nine studies (41%) with a start point in the 1990s or later. Four studies (18%) had an endpoint in the 1980s, eight (36%) studies in the 1990s, and ten (45%) in 2001 or later. Regarding the span of the period studied, the mean, median, and mode are 15 years. Seven studies considered a period of less than ten years, 12 between 10 and 20 years, and two extended over 20 years. As is shown in Table 1 and Figure 2, it is clear that the interest in demographic studies of DS increased over time.

The studies selected in this systematic review come from countries (10 studies, 45%) and country subdivisions (12 studies, 55%) from Europe (8 studies, 36%), America (6 studies, 27%), Asia (5 studies, 23%), Oceania (2 studies, 9%), and Africa (one study, 5%). Regarding cultural areas, we found 14 studies from western regions (63%), five studies from eastern Asia (22%), and two studies from Latin and Caribbean countries (9%). Four studies (18%) included ethnic groups (Chinese, Malay, Indians, White, Colored, Black, and Latino as are named in the cited sources).

### 3.1. Trends in Demographics of Down Syndrome

**Figure 3:**
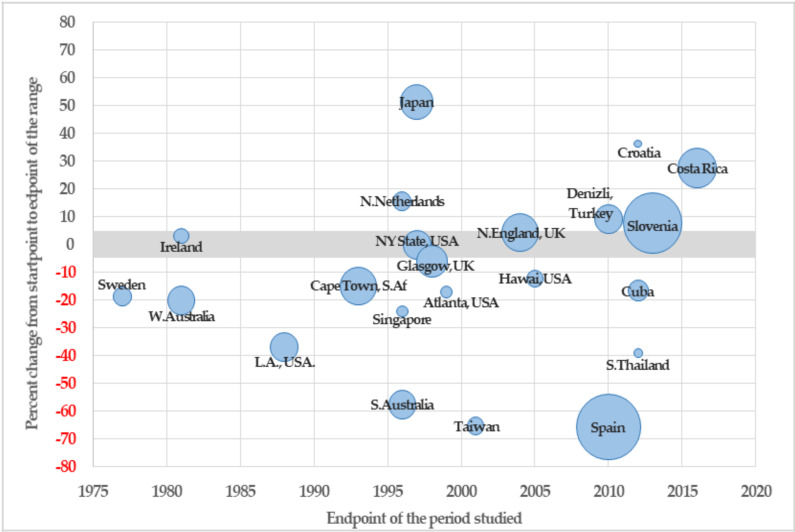
Selected studies by percent change in birthrate, region, endpoint, and time span of the period studied. Note: The area of the bubbles is proportional to the width of the period studied.

Analysis of global trends showed an overall declining trend in birthrates for the total population with DS. As is shown in Figure 3, twelve studies (55%) showed a clear decrease in birthrate, while only five studies (23%) found an increasing trend in the period studied, and five (23%) found an unclear direction.

However, this result is less clear when taking into account the time period. Following Loane [9], the trends for the most recent period (from the last 90s) showed an increasing DS birthrate, as is shown in studies from Turkey, Slovenia, Croatia, and Costa Rica [17,18,19,20]. Although at the same time, from 2010, we have found decreasing trends in studies from Spain, Thailand, and Cuba [21,22,23].

Regarding the studies with a broader study period (20 years or more), we found an increasing birthrate in Costa Rica [18] and a decreasing birthrate in Spain [21] and Cape Town [24], while Northern England [25] and Slovenia [20] show an unclear (but lightly increasing) trend.

### 3.2. Screening Tests and Prenatal Care

Global demographic trends of the DS population from 1960 to 2019 showed a consistent association between antenatal screening and a significant decrease in the incidence of DS. Ten studies [10,11,16,21,22,23,26,27,28,29] of the twelve that identified a descending birthrate determined that the spread of prenatal screening given by healthcare systems was the leading cause of this decrease.

Our results confirm that the demographical impact of antenatal screening has been clear since the 1980s, with the increasing availability of more sophisticated screening and less invasive prenatal diagnostic techniques [30]. The regions more affected by this trend are South Australia, Taiwan, and Spain [21,27,29].

However, in the results of studies from developed regions (Japan, Northern Netherlands, Northern England) with available prenatal screening for all pregnant women, the birthrate is not decreasing [25,31,32]. The improvement in healthcare is identified as a factor for the rising birthrate in Croatia, Costa Rica, Turkey, and Slovenia [17,18,19,20].

### 3.3. Related Variables: Maternal Age, Legal and Sociocultural Contexts

In the demography of DS, it is well recognized that a high incidence occurs with increasing maternal age. This trend is consistently confirmed in our review as the leading cause of the rising birthrate [18,25]. The global change of lifestyle in the last fifty years [31], with a clear tendency to postpone family planning, added to a global extension of fertility services, has resulted in an increasing pregnancy rate in patients of advanced maternal age.

Another factor influencing the rising birthrate recognized in our review is socioeconomic conditions [23], related not only with personal decisions about terminating the pregnancy but with the ability to access preventive healthcare programs and the existing legislation about medical termination of pregnancy [17].

Regarding ethnicity and sociocultural variables, we found little effect of prenatal diagnosis on birthrate in the Latino population in Los Angeles [16] and a trend to regard medical interventions on prenatal diagnosis as effected by moral sentiments in Japan [31] or religious grounds in Cape Town [24]. The variation in the prevalence of DS by ethnicity is noted, but not consistent [11].

### 3.4. Sources for Demographic Assessment of Down Syndrome

As shown in Table 1, 14 of the 22 studies selected in this review (64%) used data from registries specifically designed for epidemiological monitoring of health conditions [19]. The use of registries to conduct population-based studies is especially fitting to estimate and analyze uninterrupted data with precision. The fragmentation of registries in small (administrative) territories is an added difficulty that has been found in the sources of the studies analyzed that could be solved with global registries [22]. Without population-based data about incidence, preventive politics and health care programs will not be sufficiently effective [17].

## 4. Discussion

This review shows whether studies provided evidence to substantiate the claim that prenatal testing decreases the DS population. Our review demonstrates a consistent association between prenatal diagnosis and birthrate, enough to explain a significant fall in the prevalence of DS.

Moreover, this trend is not persistent across regions and seems to have been more robust in the 1980s and 1990s than nowadays, although it remains evident in some countries today [21,22]. It seems likely the effects of antenatal diagnosis and termination of pregnancy are less than they were decades ago in some regions [10,16,26,27,28]. It is necessary to consider that in most countries, where the decreasing trend in birthrate has been reduced or even reversed, it is much lower than it was before the massive introduction of prenatal diagnostic.

The International Convention on the Rights of Persons with Disabilities (UN, 2006) established a legal framework that directly involves this situation, based on three key articles: Article 3, which establishes non-discrimination as a general principle; Article 10, which obliges States Parties to reaffirm the inherent right to life of all human beings and to take all necessary measures to guarantee this right to persons with disabilities as to others; and Article 31, which commits States Parties to collect appropriate information, including statistical and research data, to enable them to formulate and implement disability equality policies.

It is well recognized that a high incidence of DS occurs with increasing maternal age [33], so the global trend towards increasing maternal age should expand the population of DS because of an increasing birthrate. Our findings indicate that, while this trend exists, its demographic impact is limited since it is ultimately an opposite force derived from the widespread application of prenatal testing [21]. There is a clear exception to this trend in Japan, where the increasing frequency of DS has a clear weight on the increased birthrate [31].

The improvements in healthcare systems and maternity care programs, in turn, present unclear results from a demography assessment. On the one hand, improved healthcare in some developing countries has led to higher birthrates [18,19], which are contrasted by the expansion of prenatal testing in the same regions. In conclusion, it cannot be determined that improvements in healthcare will have an impact on the demography of DS.

In short, increasing maternal age and improved survival of children with DS have offset but not inverted, the effects of antenatal screening in the declining general birthrate. The possibility of cultural, religious, ethnic, or regional differences in screening impact and maternal age-specific rates for DS should be taken into account since they are mentioned in some of the sources used, although the influence of these variables it is not deeply studied.

We found a generalized use of specific congenital birth defect registries as the primary source of data. The high prevalence of DS as a genetic cause of intellectual disability, and the increasing interest in the demographic assessment of DS, demonstrates the global need for specific registries, completed with socioeconomic variables that allow rich comparative and long-term studies.

## 5. Conclusions

After five decades of prenatal diagnosis of DS, there is an intense debate whether the widening of and improvements in genetic counseling have resulted in a relative impact on the birth prevalence of DS, shown in the rising interest in demographic studies of DS showing an increasing prevalence over time.

This study is the first systematic review of empirical studies in the demography of DS. It demonstrates that there is: (1) a consistent observation of the association between prenatal diagnosis and birthrate, enough to explain a significative decrease in the prevalence of DS; (2) a somewhat rising of incidence related to increased maternal age and extension of fertility services in healthcare systems; and (3) a generalized use of specific congenital birth defect registries as the primary source of data. Also, the influence of socio-cultural and territorial variables is mostly unclear. Our findings can inform research, policy, and practice to improve the reproductive health and quality of life of the population with DS. In short, as a general response to the main objective of this review, the impact of antenatal screening on the demography of DS is evident.

Our findings can inform research, policy, and practice to improve the reproductive health, and quality of life of the population with DS, from a demographic approach, for policymaker in disability policy, organizations on disability rights, and the healthcare system.

The policymakers need to solve the conflict between protecting disability rights and improvements on prenatal care as part of healthcare. The healthcare system needs to improve communication skills based on the related variables, i.e., pregnant women’s sociodemographic position.

A critical group of interest in this frame are the organizations of people with DS and their families, which need to incorporate demographic assessment in their programs to improve the rights, social inclusion, interpersonal relationships, and material well-being [34].

The UN Committee on the Rights of Persons with disabilities has informed that the law about the abortion discriminates against the DS population. The committee has pointed about the negative perception about disability that this law contains [15]. Although it would be theoretically possible to relate the recent stabilization in the birthrate of DS in some regions to an awareness of disability discrimination, as established by the CRPD, we have no found clear evidence of it.

Future research, based on this review, should investigate how other variables and factors impact on the demography of DS. One of these factors to be investigated are gender inequalities, especially related to care. Those studies will find the intersection between gender and disability discrimination based on CRPD.

One other hand, our review lays a way to develop the demographic assessment of DS through the use of specific congenital birth defect registries as the primary source of data. The high prevalence of DS as a genetic cause of intellectual disability, and the increasing interest in the demographic assessment of DS, demonstrates the global need for specific registries, completed with socioeconomic variables that allow comparative and long-term studies.

### Limitations

Our study had several limitations. The most important one is that the birthrate is a limited demographic indicator, and specifically in the population with DS, because we know that improvements in quality of life are extending the life expectancy, and so, implies an increase not contemplated in this work; we have done so because the birthrate is one of the few demographic indicators for which there are reliable data on DS, and there are very few regions in the world with reliable prevalence data of the whole population. Another critical limitation has to do with the scarcity of information that we offer about the influence of the termination of pregnancy laws that operate in each region; although there is literature on birthrate in DS and also literature on pregnancy termination legislation from a rights perspective, there are practically no sources that have used both views at the same time, so we can only talk by intuition in this area. A third limitation is the use of start point and endpoint of each series as an indicator; we have done this because several of the sources used primary figures at the first and last year, but not always in the center, making it impossible to create a standardized indicator or statistical test across each series. Fourthly, we made our search in English and Spanish, so some important references could be lost in other languages. Finally, we are aware that it would possibly be more accurate to use international registries that offer birth data of DS in several regions (such as EUROCAT), but it would not have allowed us to provide complementary information on the interest that this topic arouses in the recent literature, and the explanatory variables given in those sources, on the objectives of the study.

## Figures and Tables

**Table 1:** Description and main results of studies covered by the systematic review.

*N*	Study	Region	Years (Range)	Startpoint Birthrate	Endpoint Birthrate	Percent Change	Main Result (*)	Main Cause (**)	Main Source (***)
**1.**	Lindsten et al. (1981)	Sweden	1968–1977 (10)	14.6	11.9	−18.5	Descent	Screening	Registry
**2.**	Mulcahy (1983)	Western Australia	1967–1981 (15)	11.4	9.1	−20.2	Descent	Screening	Registry
**3.**	Mulcahy (1985)	Ireland	1974–1981 (8)	10	10.3	3.0	Unclear	-	Registry
**4.**	Wilson et al. (1992)	Los Angeles (USA)	1974–1988 (15)	19.0	12.0	−36.8	Descent	Screening	Record
**5.**	Molteno et al. (1997)	Cape Town (S.Africa)	1974–1993 (20)	14.2	12.1	−14.8	Descent	Unclear	Record
**6.**	Hoshi et al. (1999)	Japan	1980–1997 (18)	5.6	8.5	51.3	Ascent	Maternal Age	Registry
**7.**	Cheffins et al. (2000)	South Australia	1982–1996 (15)	9.9	4.2	−57.6	Descent	Screening	Registry
**8.**	Iliyasu (2002)	Glasgow (UK)	1980–1996 (17)	6.7	6.3	−6.0	Unclear	-	Registry
**9.**	Lai et al. (2002)	Singapore	1993–1998 (6)	11.7	8.9	−23.9	Descent	Screening	Registry
**10.**	Olsen et al. (2003)	New York State (USA)	1983–1997 (15)	9.9	9.9	0.0	Unclear	-	Registry
**11.**	Siffel et al. (2004)	Atlanta (USA)	1994–1999 (6)	12.1	10.0	−17.0	Descent	Screening	Record/Registry
**12.**	Wortelboer et al. (2004)	Northern Netherlands	1987–1996 (10)	12.8	14.8	15.6	Ascent	Maternal Age/Healthcare	Registry
**13.**	Hei-Jen et al. (2005)	Taiwan	1993–2001 (9)	4.6	1.6	−65.2	Descent	Screening	Registry
**14.**	Irving et al. (2008)	Northern England (UK)	1985–2004 (20)	11.6	12.1	4.3	Unclear	-	Survey
**15.**	McDermott et al. (2011)	Hawaii (USA)	1997–2005 (9)	9.0	7.9	−12.2	Descent	Unclear	Registry
**16.**	Acikibas et al. (2012)	Denizli (Turkey)	1996–2010 (15)	9.1	9.9	9.2	Ascent	Healthcare	Record/Survey
**17.**	Mendez-R. et al. (2014)	Cuba	2002–2012 (11)	8.4	7.0	−16.7	Descent	Screening/Maternal Age	Record
**18.**	Glivetic et al. (2015)	Croatia	2009–2012 (4)	7.4	10.1	36.3	Ascent	Unclear	Registry
**19.**	Huete-García (2016)	Spain	1976–2010 (35)	16.0	5.5	−65.6	Descent	Screening	Registry
**20.**	Gorazd et al. (2017)	Slovenia	1981–2012 (32)	5.1	5.5	7.8	Ascent	Unclear	Record
**21.**	Jarurata-nasirikul (2017)	Southern Thailand	2009–2013 (5)	9.5	5.8	−38.9	Descent	Screening/Maternal age	Survey
**22.**	Benavides (2019)	Costa Rica	1996–2016 (21)	9.1	11.6	27.5	Ascent	Maternal age/Healthcare	Registry

(*) Responding to principal objective, (**) responding to second objective, and (***) responding to third objective.

## Appendix A

The PsycINFO search is an example search strategy that reflects the terms used within each database. Subtle variations in terms arose from exploded terms as these were database specific, and the formatting varied between databases. PsycINFO 1985–present. The last run was 06/08/2020.

**Table A1:** PsycINFO search strategy.

S48	S24 and S47	Expanders—Apply equivalent subjects/Search modes—Boolean/Phrase
S47	S33 or S34 or S35 or S36 or S37 or S38 or S39 or S40 or S41 or S42 or S43 or S44 or S45	Expanders—Apply equivalent subjects/Search modes—Boolean/Phrase
S46	S9 and S32	Expanders—Apply equivalent subjects/Search modes—Boolean/Phrase
S45	AB* antenatal testing	Expanders—Apply equivalent subjects/Search modes—Boolean/Phrase
S44	TI** antenatal testing	Expanders—Apply equivalent subjects/Search modes—Boolean/Phrase
S43	TI antenatal screening	Expanders—Apply equivalent subjects/Search modes—Boolean/Phrase
S42	AB antenatal screening	Expanders—Apply equivalent subjects/Search modes—Boolean/Phrase
S41	AB prenatal screening	Expanders—Apply equivalent subjects/Search modes—Boolean/Phrase
S40	TI prenatal screening	Expanders—Apply equivalent subjects/Search modes—Boolean/Phrase
S39	AB prenatal screening	Expanders—Apply equivalent subjects/Search modes—Boolean/Phrase
S38	AB antenatal diagnosis	Expanders—Apply equivalent subjects/Search modes—Boolean/Phrase
S37	TI antenatal diagnosis	Expanders—Apply equivalent subjects/Search modes—Boolean/Phrase
S36	TI prenatal testing	Expanders—Apply equivalent subjects/Search modes—Boolean/Phrase
S35	AB prenatal testing	Expanders—Apply equivalent subjects/Search modes—Boolean/Phrase
S34	TI prenatal diagnosis	Expanders—Apply equivalent subjects/Search modes—Boolean/Phrase
S33	AB prenatal diagnosis	Expanders—Apply equivalent subjects/Search modes—Boolean/Phrase
S32	S26 or S27 or S28 or S29 or S30 or S31	Expanders—Apply equivalent subjects/Search modes—Boolean/Phrase
S31	antenatal testing	Expanders—Apply equivalent subjects/Search modes—Boolean/Phrase
S30	antenatal diagnosis	Expanders—Apply equivalent subjects/Search modes—Boolean/Phrase
S29	antenatal screening	Expanders—Apply equivalent subjects/Search modes—Boolean/Phrase
S28	prenatal screening	Expanders—Apply equivalent subjects/Search modes—Boolean/Phrase
S27	prenatal testing	Expanders—Apply equivalent subjects/Search modes—Boolean/Phrase
S26	prenatal diagnosis	Expanders—Apply equivalent subjects/Search modes—Boolean/Phrase
S25	S14 n1 S23	Expanders—Apply equivalent subjects/Search modes—Boolean/Phrase
S24	S14 and S23	Expanders—Apply equivalent subjects/Search modes—Boolean/Phrase
S23	(S15 or S16 or S17 or S18 or S19 or S20 or S21 or S22)	Expanders—Apply equivalent subjects/Search modes—Boolean/Phrase
S22	TI prevalence	Expanders—Apply equivalent subjects/Search modes—Boolean/Phrase
S21	AB prevalence	Expanders—Apply equivalent subjects/Search modes—Boolean/Phrase
S20	AB incidence	Expanders—Apply equivalent subjects/Search modes—Boolean/Phrase
S19	TI incidence	Expanders—Apply equivalent subjects/Search modes—Boolean/Phrase
S18	TI demographic characteristics	Expanders—Apply equivalent subjects/Search modes—Boolean/Phrase
S17	AB demographic characteristics	Expanders—Apply equivalent subjects/Search modes—Boolean/Phrase
S16	AB epidemiology	Expanders—Apply equivalent subjects/Search modes—Boolean/Phrase
S15	TI epidemiology	Expanders—Apply equivalent subjects/Search modes—Boolean/Phrase
S14	S10 or S11 or S12 or S13	Expanders—Apply equivalent subjects/Search modes—Boolean/Phrase
S13	AB down’s syndrome	Expanders—Apply equivalent subjects/Search modes—Boolean/Phrase
S12	TI down’s syndrome	Expanders—Apply equivalent subjects/Search modes—Boolean/Phrase
S11	TI trisomy 21	Expanders—Apply equivalent subjects/Search modes—Boolean/Phrase
S10	AB trisomy 21	Expanders—Apply equivalent subjects/Search modes—Boolean/Phrase
S9	S7 and S8	Expanders—Apply equivalent subjects/Search modes—Boolean/Phrase
S8	S3 or S4 or S5 or S6	Expanders—Apply equivalent subjects/Search modes—Boolean/Phrase
S7	S1 or S2	Expanders—Apply equivalent subjects/Search modes—Boolean/Phrase
S6	prevalence	Expanders—Apply equivalent subjects/Search modes—Boolean/Phrase
S5	incidence	Expanders—Apply equivalent subjects/Search modes—Boolean/Phrase
S4	demography	Expanders—Apply equivalent subjects/Search modes—Boolean/Phrase
S3	epidemiology	Expanders—Apply equivalent subjects/Search modes—Boolean/Phrase
S2	trisomy 21	Expanders—Apply equivalent subjects/Search modes—Boolean/Phrase
S1	down’s syndrome	Expanders—Apply equivalent subjects/Search modes—Boolean/Phrase
